# Comparative inactivation of *Listeria monocytogenes* in human and bovine milk treated with high-pressure processing and UV-C treatment and potential growth during refrigerated shelf-life

**DOI:** 10.3389/fmicb.2026.1774372

**Published:** 2026-04-08

**Authors:** Hussein M. H. Mohamed, Ningjian Liang, Austin Lowder, David C. Dallas, Joy Waite-Cusic

**Affiliations:** 1 Department of Food Science and Technology, Oregon State University, Corvallis, OR, United States; 2 Nutrition Program, College of Health, Oregon State University, Corvallis, OR, United States; 3 Department of Animal, Veterinary and Food Sciences, University of Idaho, Moscow, ID, United States; 4 JBT Avure Technologies, Erlanger, KY, United States

**Keywords:** matrix effects, milk type, non-thermal processing, optical properties, pasteurization

## Abstract

Human and bovine milk differ in composition, including the concentration of antimicrobial components, which may impact the efficacy of non-thermal processes to inactivate foodborne pathogens and may also impact the growth of pathogens introduced via post-processing contamination during refrigerated storage. Two strains of *Listeria monocytogenes* (ATCC 35152 and OSY-328) were inoculated into human and bovine milk and treated with high-pressure processing (HPP; 400, 450, and 500 MPa for 1, 5 and 9 min) or ultraviolet-C (UV-C) irradiation (500–7,300 J/L). Differences in HPP and UV-C treatment efficacy between the two milk types were evaluated. In addition, the growth of *L. monocytogenes* inoculated into Holder pasteurized (HoP, 62.5 °C, 30 min), HPP-treated (500 MPa, 9 min, 16–19 °C) and UV-C-treated (5,000J/L) human and bovine milk were studied during storage at 4 °C for 22 days. Identical processing parameters for either HPP or UV-C resulted in greater reductions of both *L. monocytogenes* strains in human milk compared to bovine milk. Sufficient lethality (>5-log CFU/mL reduction) of inoculated *L. monocytogenes*, which is the target for pasteurization, was achieved when raw human milk was treated with HPP at 500 MPa for 5 and 9 min and UV-C at ≥5,000 J/L; however, a 5-log reduction was not achieved at any of the tested parameters tested in bovine milk. Across storage at 4 °C of human milk treated with HoP, HPP or UV-C, and then inoculated with *L. monocytogenes,* the counts of the two microorganisms decreased and reached less than the detectable limit (1 CFU/mL) after 18–20 days. However, for bovine milk under the same conditions, *L. monocytogenes* counts increased, reaching >7 log CFU/mL after 22 days. Overall, *L. monocytogenes* is more sensitive to HPP and UV-C processing in human milk than in bovine milk, and human milk displayed inherent antimicrobial activity towards *L. monocytogenes*, even after processing, whereas bovine milk supported growth of *L. monocytogenes* during refrigerated storage.

## Introduction

Milk is a complex biological fluid which is a rich source of nutritional components such as protein, fat, carbohydrates, lactose, vitamins and minerals, as well as an array of bioactive compounds, such as antimicrobial proteins and peptides. Milk has been reported to provide other health benefits, including protection against infection (Morrow and Rangel, 2004), enhancing gastrointestinal tract function (Hernell and Bläckberg, 1994; Tong et al., 2020), improving bone health (Wallace et al., 2021; Sharifan et al., 2025) and increasing blood levels of essential minerals and vitamins (Górska-Warsewicz et al., 2019). Milk components vary greatly across species (Roy et al., 2020). Bovine and human milk, in particular, have many compositional differences; human milk contains lower concentrations of total protein, immunoglobulin G, lactoperoxidase and minerals, and higher amounts of lactose, fat, immunoglobulin A, lactoferrin, lysozyme and glycerol monolaurate (GML) compared with bovine milk (Hettinga et al., 2011; Schlievert et al., 2019; Liu et al., 2021; Sindi et al., 2025). Among these, lactoperoxidase, lactoferrin, lysozyme and GML have been reported to have antimicrobial properties (Masschalck and Michiels, 2003; Seifu et al., 2005; González-Chávez et al., 2009; Schlievert et al., 2019). These compositional differences in bovine and human milk could impact their degree of antimicrobial activity.

Milk supplies mammalian young with a nutritious and functional source of food; however, it is also highly susceptible to contamination with organisms that can cause infection. This issue is less of a concern for direct fed offspring but is critical to the safety of milk products with widespread distribution, particularly for products that will be fed to the most vulnerable of the human population (i.e., infants in neonatal intensive care). Thermal pasteurization serves as the current standard processing approach for human and bovine milk to reduce the risk of foodborne pathogen transmission. Existing thermal pasteurization processes for bovine milk are designed to achieve a 5-log reduction of the most resistant non-sporulating pathogen likely to be present (i.e., *Coxiella burnetti*) (Hudson et al., 2003). Human milk banks adopted bovine milk pasteurization treatments for human milk processing which have since been demonstrated to be sufficient to control human milkborne pathogens (O'Coonor et al., 2015). There is interest in validating non-thermal processing methods (e.g., high-pressure processing (HPP) and ultraviolet-C (UV-C)) that will achieve safety goals (i.e., 5-log reduction of relevant pathogens) while also improving the preservation of the bioactive components found in raw milk.

The most common thermal processing treatment for enhancing the microbial safety of donor human milk in human milk banks and bovine milk at small-scale dairies is Holder pasteurization (HoP) (treatment at 62.5 °C for 30 min), also referred to as vat or low temperature-long time (LTLT) pasteurization (Peila et al., 2017; Wesolowska et al., 2019). HoP is effective at inactivating the relevant vegetative pathogens (Chen and Allen, 2001; Hamprecht et al., 2004; Landers and Updegrove, 2010; Wesolowska et al., 2019); however, thermal pasteurization also decreases the concentration and/or activity of some bioactive proteins (e.g., immunoglobulins (IgA, IgG, IgM), lactoferrin), enzymes (e.g., lipase, lysozyme, alkaline phosphatase), and hormones (e.g., adiponectin, insulin) that may promote immune function and infant growth and development (Hamprecht et al., 2004; Czank et al., 2009; Baro et al., 2011; Ley et al., 2011; Arslanoglu et al., 2013; Liang et al., 2023, 2024).

HPP uses a liquid medium to transduce very high-pressures (typically 200–700 MPa) to a food product in flexible packaging for typically short durations (a few seconds to several minutes); however, most commercial equipment operates under a maximum pressure of < 600 MPa. HPP inactivates bacteria by inducing a loss of membrane functionality from discrepant compression of bacterial membrane proteins and the lipid bilayer, which disrupts pH and ion gradients necessary for cell function (Sehrawat et al., 2021). There is also evidence of HPP treatment causing ribosome dissociation (Niven et al., 1999; Maldonado et al., 2015) and oxidative stress in bacteria (Aertsen et al., 2005). The efficacy of HPP depends on the pressure, time under pressure, holding temperature, and number of pressurization cycles (Chawla et al., 2011; Georget et al., 2015; Muntean et al., 2016; Liang et al., 2023; Sykora et al., 2025). The composition of the food matrix will also influence the efficacy of HPP against microorganisms. In milk, studies have shown that optimizing these parameters can lead to significant reductions in common spoilage and pathogenic organisms such as *Salmonella* spp., *Listeria monocytogenes* and *Escherichia coli* (Chawla et al., 2011). At some treatment parameter combinations, HPP treatment of human milk can provide > 5-log reductions of vegetative bacterial pathogens while better preserving key bioactive components (e.g., IgA, IgM, lactoferrin, bile salt-stimulated lipase) compared with HoP (Liang et al., 2023).

UV-C light (200–280 nm wavelength) can also inactivate microbes in milk. UV-C exposure leads to cross-linking of nucleic acids in DNA and RNA molecules which prevents them from being replicated and ultimately causes cell death (Hijnen et al., 2006). UV-C has long been used as a treatment strategy for the disinfection of drinking water as its efficiency is a function of light transmission. The opacity due to suspended solids in milk limits the penetration of UV-C light through the matrix and its antimicrobial efficacy (Antonio-Gutiérrez et al., 2019); however, processing systems incorporating mixing and/or reducing liquid depth to maximize surface area exposure are effective strategies to overcome this limitation (e.g., Franz et al., 2009; Gok, 2021; Singh et al., 2025; Vashisht et al., 2022). Specific dosages of UV-C irradiation of human milk have been shown to provide > 5-log reductions of vegetative bacterial pathogens, while better preserving the bioactive proteins IgA, IgG, IgM, lactoferrin, lysozyme and bile salt-stimulated lipase (Liang et al., 2024).

We hypothesized that due to differences in composition, particularly the differences in the concentration of antimicrobial components, HPP and UV-C doses required to achieve equivalent reductions of foodborne pathogens would differ between human and bovine milks. We also hypothesized that these treatments could lead to differences in the retention of antimicrobial components within these milks and impact the behavior of pathogens during the shelf-life of the processed product. To test these hypotheses, *Listeria monocytogenes* was selected as the target pathogen due to its critical importance as a neonatal pathogen (high case fatality), demonstrated routes of contamination in mammalian milk (Poulsen et al., 2013; Charlier et al., 2023), its relative resistance to processing (Lado and Yousef, 2002; Benlloch-Tinoco et al., 2014; Bucur et al., 2018), and its ability to grow in products during refrigerated storage (Strydom and Witthuhn, 2015; Osek et al., 2022). Growth in processed products is a relevant consideration due to the opportunity for post-processing contamination in systems where the lethal process occurs prior to packaging.

Therefore, the objectives of this study were to 1) compare the inactivation of *L. monocytogenes* in human and bovine milk when treated with HPP and UV-C; and 2) to evaluate the behavior of *L. monocytogenes* in human and bovine milk treated with HoP, HPP and UV-C to evaluate treatment impact on antimicrobial activity during storage.

## Materials and methods

### Experimental design

**Figure 1:**
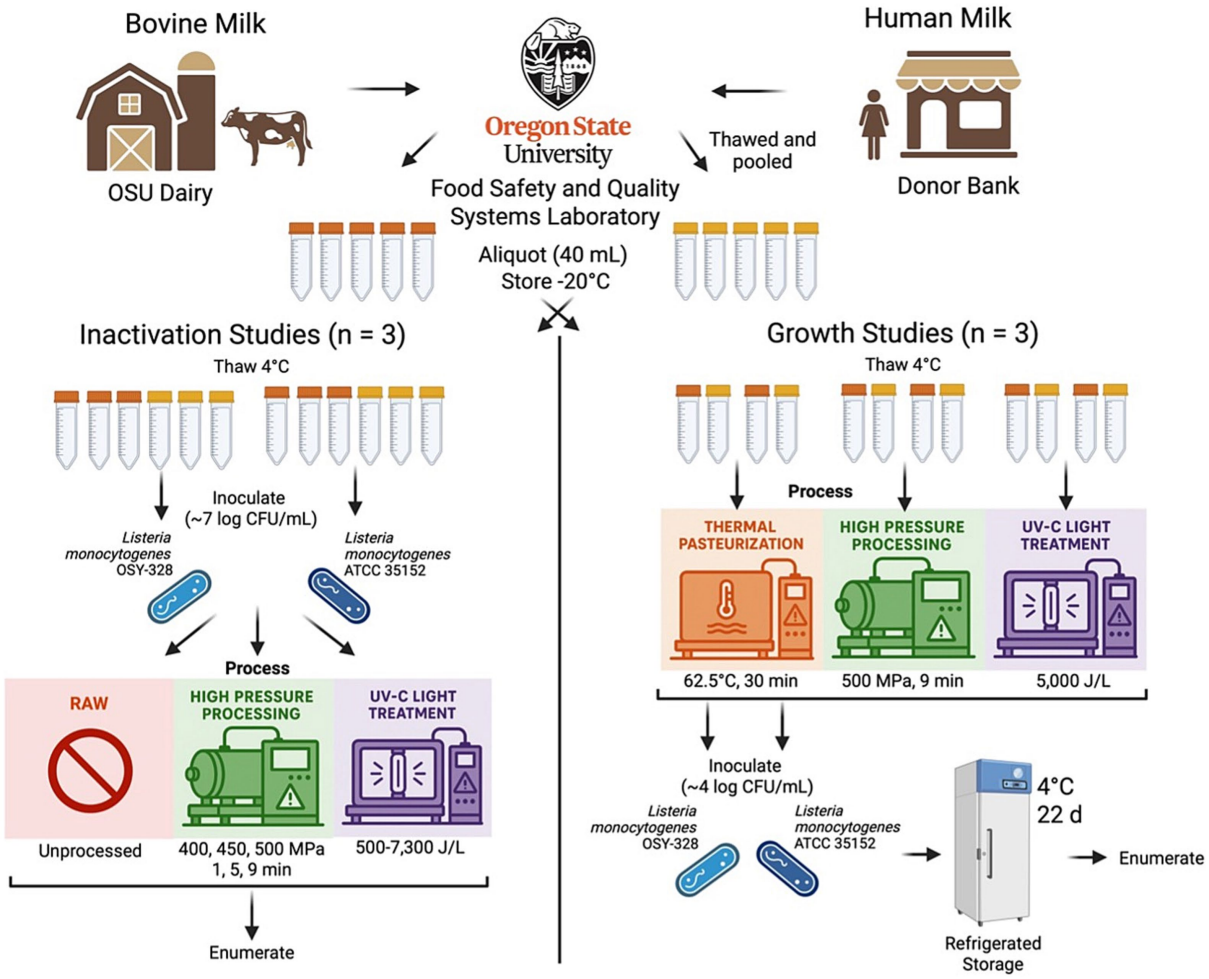
Overall experimental design to evaluate the impact of high-pressure processing (HPP) and ultraviolet-C (UV-C) processing treatments of bovine and human milk on the behavior of *Listeria monocytogenes* OSY-328 and ATCC 35152. Created in BioRender (https://BioRender.com/5d5s5id; clipart images of processing treatments were generated using ChatGPT).

The overall experimental design is shown in Figure 1. This research was conducted in two stages: inactivation studies and growth studies. One collection from each milk source (described below) was used for the entire project. Each study type was performed in triplicate with independently prepared inoculants used for each replication. Inactivation studies were focused on HPP and UV-C processing impacts using raw (unprocessed) milk as the control. Growth studies were performed in milks treated with selected HPP and UV-C treatments with milks treated with thermal pasteurization (HoP) as the control. Raw milk samples were not used for growth studies due to enumeration challenges of the background microbiota.

### Raw milk sources and handling

Frozen, unprocessed donor human milk (DHM) was obtained from the Northwest Mothers Milk Bank (Portland, OR). The DHM was collected and frozen in the homes of various donors and later donated to the milk bank over a period of time. Multiple bags of individual frozen milk donations (240 mL/bag) were transported on ice to Oregon State University and stored at −20 °C until use. Frozen human milk bags were thawed at 4 °C for 48 h and human milk from all bags were pooled, mixed, distributed into 40 mL aliquots and stored at −20 °C until use. Pooling of human milk was necessary to eliminate confounding effects due to milk composition differences between donors; this pooling process also resulted in the two milk types being more comparable and representative of the milking population (many individuals contributing to the bulk samples). Unprocessed (raw) bovine milk was obtained from the Oregon State University dairy farm (Corvallis, OR). Raw milk was collected from the bulk silo after the morning milking and transported (<2 km) via automobile in sanitized stainless steel milk cans to the Food Safety and Quality Systems Laboratory (Corvallis, OR). Raw milk was subdivided into 40 mL aliquots, and stored at −20 °C until use (up to 12 months). Frozen human and bovine milk samples (40 mL) were thawed at 4 °C for 24 h before use in experiments.

### Bacterial strains and inoculum preparation

Two *L. monocytogenes* strains were selected for use in this study based on prior reports on increased piezotolerance (OSY-328) (Chung et al., 2005; Waite and Yousef, 2008) or their status as a proposed type strain (ATCC 35152) (Jones and Seeliger, 1983). *L. monocytogenes* OSY-328 was provided by Dr. Ahmed Yousef’s food safety laboratory at The Ohio State University (Columbus, OH) and *L. monocytogenes* ATCC 35152 was purchased from ATCC (Manassas, VA). Both strains were cryopreserved at −80 °C in 35% glycerol in tryptic soy broth (TSB; Lansing, MI). Each strain was revived by inoculating into tryptic soy broth (TSB; Neogen, Lansing, MI) supplemented with 0.6% yeast extract (YE, Neogen, Lansing, MI) (TSBYE) and incubating at 37 °C for 48 h. Strains were verified for purity and identity by streaking for isolation on Harlequin *Listeria* chromogenic agar (HQ; Neogen, Lansing, MI) with incubation at 37 °C for 48 h. A single, typical (blue-green colonies with opaque halo) isolated colony was transferred from HQ agar to 10 mL of TSBYE and incubated at 37 °C for 48 h to reach ~8 log CFU/mL. The TSBYE culture of each strain was centrifuged at 7,500 *x g* for 10 min at 15 °C and the pellet was resuspended into 10 mL of human or bovine milk. An aliquot (100 μL) of the bacterial milk suspension was used to inoculate raw milk to obtain a cell density of ~7 log CFU/mL for inactivation studies (described below). The bacterial milk suspension was further serially diluted in human or bovine milk to an approximate cell density of 5 log CFU/mL and an aliquot (100 μL) was used to inoculate thermally treated (HoP), HPP, or UV-C treated milk to achieve a final cell density of ~4 log CFU/mL for growth studies (described below).

### Inactivation studies—high-pressure processing (HPP)

Human and bovine milk samples inoculated with each *Listeria* strain were aliquoted (2 mL) into high barrier bags (12 cm x 18 cm, Vacuum Sealers Unlimited, CA) and vacuum sealed. Samples were shipped overnight on dry ice to Avure Food Lab (Erlanger, KY). Frozen milk samples were received at the HPP facility, thawed under cold running water for 30 min, and held at 4 °C for up to 4 h prior to HPP treatment on the same day. Samples were processed using the Avure HPP Food Machine AV-10 (Avure Technologies, Middletown, OH) at the following pressure–time combinations: 400, 450 and 500 MPa for 1, 5 and 9 min (9 treatments total). Pressure was transduced through cold water (2–6 °C) which resulted in a holding temperature of 16–19 °C due to adiabatic heating. Pressurization and depressurization times increased with increasing pressure and ranged from 49–78 s (pressurization) and 11–15 s (depressurization), respectively. Processed samples were immediately returned to 4 °C after processing. Untreated travel controls were thawed and held at 4 °C until all samples were processed. Processed and control milk samples were packed in dry ice and shipped to Oregon State University (Corvallis, OR) via next day air. Upon arrival, samples were thawed at room temperature for 30 min prior to microbial analysis.

### Inactivation studies—ultraviolet-C (UV-C) treatment

Inoculated milk (120 mL each, 4 °C) was transferred into a 150 mL sterile beaker along with a magnetic stir bar and placed on a stir plate. A 254 nm UV-C light source (Rexim LLC, Part #143 MHFUV-H9WG23) was submerged into the beaker and magnetic stirring was applied at 400 rpm and covered with aluminum foil. A milk sample (1 mL) was removed from the beaker every 60–120 s with corresponded to UV-C doses of 500–7,300 J/L which was used for microbial analysis. The UV-C lamp had an output power of 1 W at the middle of the lamp. The dosage of UV-C (J/L) was calculated by multiplying the output power of the UV-C (W) and treatment time (s) and dividing the result by the sample volume (L) (e.g., 1 W x 60 s/0.12 L). Therefore, the estimated dosages at each time point ranged from 500 J/L (1 min) to 7,300 J/L (14 min) of exposure to UV-C. The calculated dose was corrected for the cumulative removal of 1 mL samples at specified time intervals (9 mL total). The entire UV-C treatment was repeated on three separate days using independently prepared inocula.

### Growth studies—processed milk preparation (HoP, HPP, UV-C)

Thawed human and bovine milk samples were divided into 3 groups. One group was thermally processed to meet Holder pasteurization standards (HoP; 62.5 °C for 30 min) in a circulating thermostatic water bath (Anova Inc., USA). Come-up time (CUT) was approximately 10 min. The second and third groups were treated with UV-C at 5,000 J/L and HPP at 500 MPa for 9 min, respectively, as described above. The treated milk samples (40 mL) of each group were inoculated with individual *L. monocytogenes* strains (ATCC 35152 or OSY 328) to achieve a target cell density of ~4 log CFU/mL (described in inoculum preparation section above). Inoculated milk samples were stored at 4 °C for 22 d and sampled every 2 days for microbial analysis.

### Microbial analysis—enumeration of *Listeria monocytogenes*

Milk samples spread plated directly (1 mL or 100 μL) and were serially diluted in 0.1% peptone water (100 μL:900 μL) prior to spread-plating (100 μL) on tryptic soy agar with 0.6% yeast extract (TSAYE; Neogen). Colonies were counted following incubation at 37 °C for 48 h. The detection limit for the plating scheme was 1 CFU/mL. For statistical purposes, samples with no detectable colonies were assigned a value of 1 CFU on the lowest dilution plated (i.e., 1 CFU/mL). Plate counts of each sample were transformed to log CFU/mL.

### Data analysis

JMP Pro version 18 (SAS Institute, Cary, NC) was used for all data visualization and statistical analyses. For inactivation studies, microbial reductions were calculated by subtracting the cell density (log CFU/mL) of the treated sample (survivors) from the cell density (log CFU/mL) of the control (inoculated milk; no HPP treatment) for each replicate. For HPP inactivation studies, reductions were compared using a mixed model analysis of variance (ANOVA) with milk type, *L. monocytogenes* strain, replicate, pressure, time, and pressure*time as fixed discrete effects. For UV-C inactivation studies, reductions were compared using a mixed model ANOVA with UV-C dose as a continuous variable and milk type, *L. monocytogenes* strain, and replicate as fixed discrete effects. Tukey’s HSD was used as the post-hoc analysis. One-sided, one-sample t-tests were performed for selected HPP and UV-C treatments to determine whether treatments achieved a > 5-log reduction of each *L. monocytogenes* strain in human milk (*p-*value < 0.05).

For growth studies, changes in cell density were calculated by subtracting the log-transformed plate counts for each storage timepoint from its corresponding control (time 0). Slight differences in starting cell densities (3.77–4.38 log CFU/mL) had an unintended impact on the scale of change which were bound by the maximum cell density (8.32 CFU/mL) and the detection limit of the plating scheme (1 CFU/mL). Therefore, changes in cell density were normalized to facilitate comparisons between treatments. Normalization for bovine milk samples was performed by multiplying each value by the ratio of the maximum cell density increase for the entire data set (4.79 log CFU/mL) to the maximum cell density increase for each replicate. Normalization of human milk samples was performed by multiplying each value by the ratio of the maximum cell density decrease for the entire set (4.23 log CFU/mL) to the maximum cell density decrease for each replicate. *L. monocytogenes* behavior in processed milk during refrigerated storage (change in cell density; log CFU/mL) was analyzed using a mixed model ANOVA for each milk type with storage time as a continuous variable and treatment, replicate, *L. monocytogenes* strain, and treatment*strain as fixed variables. Tukey’s HSD was used as the post-hoc test to identify statistical differences in *L. monocytogenes* cell densities during storage as a result of processing treatments (*p*-value < 0.05).

## Results

### *Listeria monocytogenes* inactivation by HPP in human and bovine milk

**Figure 2:**
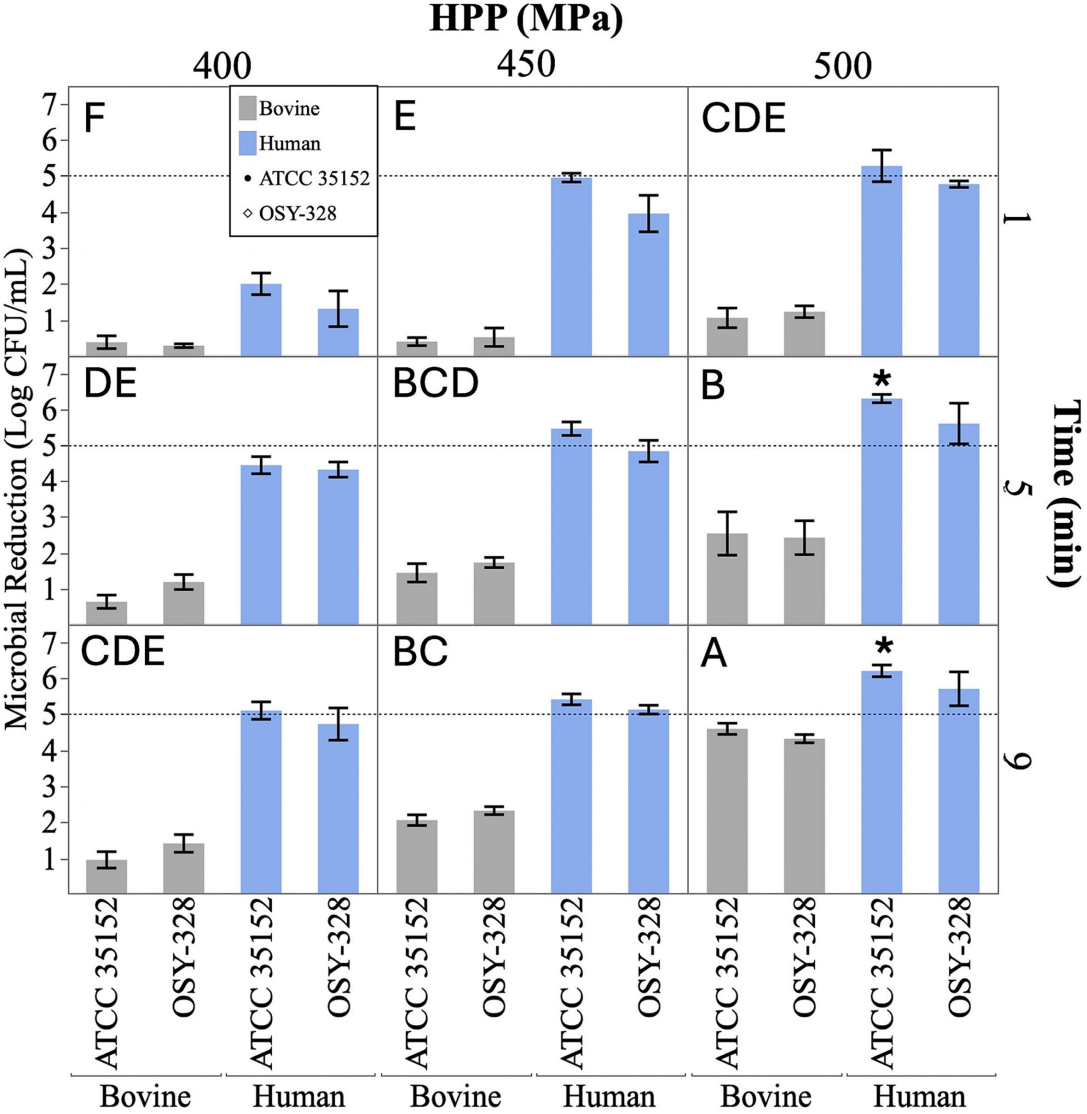
Microbial reductions (log CFU/mL) of *Listeria monocytogenes* ATCC 35152 and *Listeria monocytogenes* OSY-328 in bovine (gray) and human (blue) milk treated with high-pressure processing (HPP: 400, 450, and 500 MPa for 1, 5, and 9 min). Data are represented as the mean ± standard error (*n* = 3 independent replicates). The dashed line indicates the targeted 5-log reduction. There was a significant difference in the efficacy of all HPP treatments between milk types (two-sided, two-sample *t*-tests; *p-*values < 0.003). Pressure–time combinations that do not share a capital letter in the upper left-hand corner significantly differ in their total efficacy of reducing *L. monocytogenes* in milks (Mixed Model ANOVA with Tukey’s HSD post-hoc; *p*-value < 0.05). An asterisk (*) indicates treatments that confidently achieved a > 5-log CFU/mL reduction (one-sided, one-sample *t*-test; *p*-value < 0.05).

The efficacy of HPP (400–500 MPa, 1–9 min) at reducing *L. monocytogenes* ATCC 35152 and OSY-328 populations in human and bovine milk are shown in Figure 2. As expected, increasing pressure and increasing time supported greater levels of inactivation of both strains in both milk types (mixed model ANOVA; *p-*values < 0.0001). The two *L. monocytogenes* strains did not significantly differ in their sensitivity to HPP treatment in milk systems (*p*-value = 0.146). However, HPP treatment resulted in significantly greater inactivation of both *L. monocytogenes* strains in human milk than in bovine milk at the pressure–time combinations tested (*p-*value < 0.0001). Differences in HPP efficacy between milk type ranged from a 1.32-log reduction (400 MPa, 1 min) to 3.99-log reduction (450 MPa, 1 min).

Two of the tested HPP treatments (500 MPa, 5 min and 500 MPa, 9 min) were able to confidently achieve a > 5-log reduction of *L. monocytogenes* ATCC 35152 in human milk (one-sided, one-sample t-tests; *p-*values < 0.01). While these same treatments achieved an average log reduction of > 5-log CFU/mL of *L. monocytogenes* OSY-328 in human milk (5.60- and 5.70-log reduction, respectively), statistical analysis did not confirm that this treatment would achieve the targeted > 5-log reduction with sufficient confidence (95%) for process determination (one-sided, one-sample t-tests; *p*-values > 0.13). None of the HPP treatments of bovine milk were able to confidently achieve the 5-log reduction pasteurization standard for either of the *L. monocytogenes* strains. Maximum reductions in bovine milk were accomplished at the most extreme treatment (500 MPa, 9 min) at 4.59 ± 0.27 log CFU/mL and 4.32 ± 0.19 for *L. monocytogenes* ATCC 35152 and OSY-328, respectively.

### *Listeria monocytogenes* inactivation by UV-C in human and bovine milk

**Figure 3:**
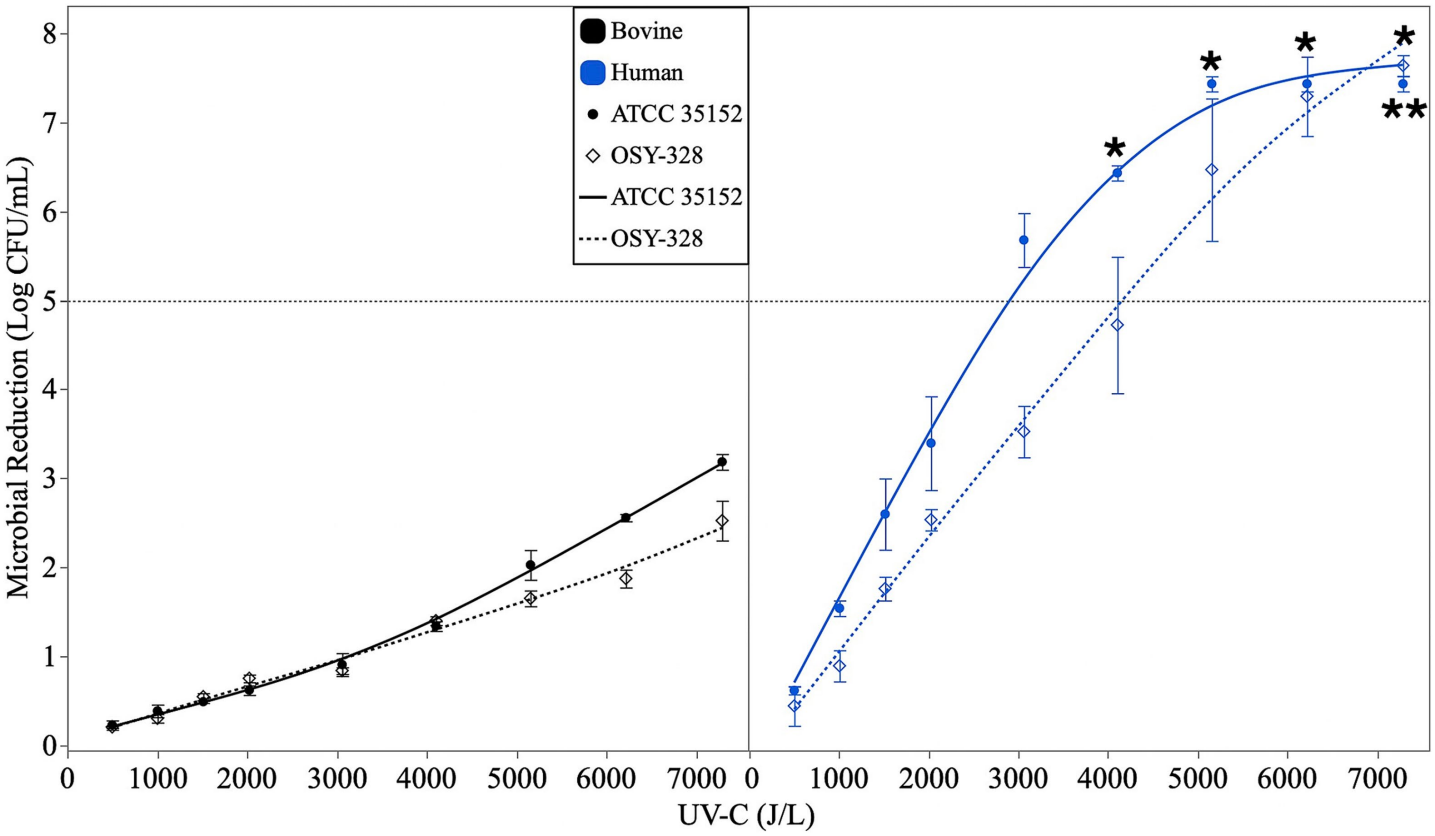
Microbial reductions (log CFU/mL) of *Listeria monocytogenes* ATCC 35152 and *Listeria monocytogenes* OSY-328 in bovine milk (left) and human milk (right) treated with ultraviolet-C irradiation (UV-C) at doses ranging from 500 to 7,300 J/L. Data are represented as the mean ± standard error (*n* = 3 independent replicates). Asterisks indicate treatments that achieved a > 5-log CFU/mL reduction of *L. monocytogenes* ATCC 35152 (*) or *L. monocytogenes* OSY-328 (**) (one-way, one-sample *t*-tests; *p*-value < 0.05).

The efficacy of UV-C treatment (500–7,300 J/L) at reducing *L. monocytogenes* ATCC 35152 and OSY-328 populations in human and bovine milk are shown in Figure 3. As expected, *L. monocytogenes* reduction due to UV-C treatment was dose-dependent (mixed model ANOVA; *p-*value < 0.0001). UV-C inactivation was significantly more effective in human milk than in bovine milk (*p*-value < 0.0001). *L. monocytogenes* ATCC 35152 was significantly more sensitive to UV-C treatment in both milk types than *L. monocytogenes* OSY-328 (*p-*value = 0.0157).

All UV-C treatments of at least 4,000 J/L achieved a > 5-log reduction of *L. monocytogenes* ATCC 35152 in human milk (one-sided, one-sample t-tests; *p-*values < 0.0018), whereas more intense UV-C treatments (at least 6,000 J/L) were required to confidently achieve a > 5-log reduction of *L. monocytogenes* OSY-328 in human milk (one-sided, one-sample *t*-tests; *p-*values < 0.0179). None of the UV-C treatments tested approached the 5-log reduction target of *L. monocytogenes* in bovine milk. The highest UV-C dose (7,300 J/L) applied to bovine milk resulted in reductions of 3.19 ± 0.15 and 2.53 ± 0.39 log CFU/mL of *L. monocytogenes* ATCC 35152 and OSY-328, respectively.

### *Listeria monocytogenes* behavior in processed human and bovine during refrigerated storage

**Figure 4:**
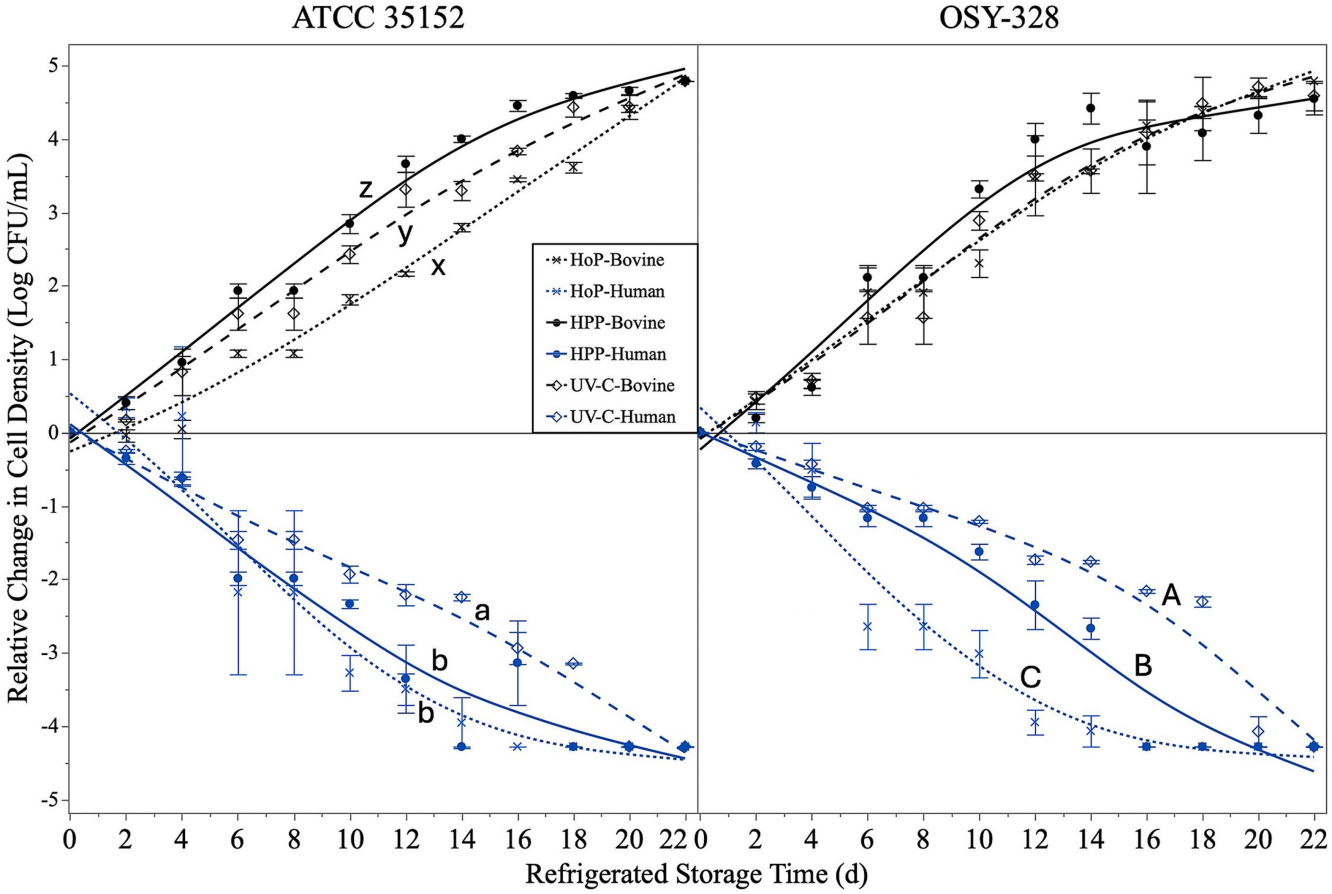
Change in relative cell density (log CFU/mL) of *Listeria monocytogenes* ATCC 35152 (left) and *L. monocytogenes* OSY-328 (right) inoculated into bovine milk (top) or human milk (bottom) after processing with Holder pasteurization (HoP; 62.5 °C, 30 min), high-pressure processing (HPP; 500 MPa, 9 min), or ultraviolet-C irradiation (UV-C; 5,000 J/L) during refrigerated storage (4 °C, 22 days). Data are represented as the mean ± standard error (*n* = 3 independent replicates) with the lines representing the smooth fit of the data for each *L. monocytogenes* strain in each processed milk type. Processed milk types that do not share a common letter differ in the behavior of *L. monocytogenes* during refrigerated storage (Mixed Model ANOVA with Tukey’s HSD *post-hoc*; *p*-value < 0.05; *n* = 3 independent replicates).

The behavior of *L. monocytogenes* strains in human and bovine milk during refrigerated storage is shown in Figure 4. Both *L. monocytogenes* strains were capable of substantial growth (>4-log CFU/mL growth by 22 days) in bovine milk that was previously processed by HoP, HPP, and UV-C treatments. In contrast, neither of the *L. monocytogenes* strains could grow in processed human milk and significantly decreased (~4-log CFU/mL reduction) in cell density during refrigerated storage.

The behavior of *L. monocytogenes* was impacted by the prior processing treatments of both bovine and human milk; however, the degree of impact differed based on milk type and *L. monocytogenes* strain (Figure 4). The relative growth of *L. monocytogenes* ATCC 35152 was significantly impaired in HoP-treated bovine milk compared with UV-C-treated or HPP-treated bovine milk (*p-*value < 0.05). The growth of this strain in UV-C treated bovine milk was comparable with HPP-treated bovine milk (*p-*value < 0.05). In contrast, the growth of *L. monocytogenes* OSY-328 in bovine milk was unaffected by previous processing treatments (*p-*value = 0.575). The relative die-off of both *L. monocytogenes* strains in human milk was significantly influenced by prior processing. Both strains showed significantly enhanced survival in human milk that was previously treated with UV-C compared with the other treatments (*p*-value < 0.05). *L. monocytogenes* OSY-328 displayed significantly enhanced survival in human milk previously treated with HPP compared to human milk previously treated with HoP (*p*-value < 0.05), whereas there was no significant difference in the survival of *L. monocytogenes* ATCC 35152 in human milk previously treated with HPP or HoP (*p-*value > 0.05).

## Discussion

To our knowledge, this study provides the first direct comparison of HPP and UV-C processing methods on the inactivation *L. monocytogenes* in human versus bovine milk. While we hypothesized that these nonthermal processing treatments would be more effective in human milk than bovine milk, the differences in process efficacy were more substantial than anticipated. While human and bovine milk have comparable macronutrient composition, they differ substantially the quantity of components with known or suggested antimicrobial activity, including lactoferrin, lysozyme, several immunoglobulins and GML (Hettinga et al., 2011). Human milk contains endogenous antimicrobial peptides like human beta-defensin 1 (Jia et al., 2001), human beta-defensin 2 (Wang et al., 2014) and cathelicidins (Murakami et al., 2005). Bovine milk contains beta-defensin 2 and 3 (Tomasinsig et al., 2010; Smolenski et al., 2011), lingual antimicrobial peptide (Isobe, 2017) and cathelicidins (Addis et al., 2016). There is a lack of literature that directly compares the abundance and antimicrobial activity of defensins and cathelicidins from human and bovine milk. Both bovine and human milk also contain an array of peptides derived from the action of endogenous proteases (e.g., plasmin, elastase) on milk proteins, some of which have antimicrobial activity (Dallas et al., 2013; Guerrero et al., 2014; Dallas et al., 2015; Sedaghati et al., 2016; Larsen et al., 2021). The abundance and antimicrobial activity of human and bovine milk protein-derived endogenous milk peptides have not been compared. Differences in the presence and abundance of such peptides could also play a role in the observed differential sensitivity of these milks to processing-induced pathogen inactivation.

High-pressure processing applied at 500 MPa for 5 and 9 min achieved the inactivation target (>5-log reduction) of *L. monocytogenes* ATCC 35152 in human milk with 95% statistical confidence; however, this level of statistical confidence was not obtained for *L. monocytogenes* OSY-328. *L. monocytogenes* OSY-328 was selected due to previous reports of its piezotolerance (Chung et al., 2005; Waite and Yousef, 2008); however, this is the first study to directly compare these two strains in response to HPP. Collectively, our results found no significant difference in the piezotolerance of these two strains in either milk under the HPP conditions tested; however, the ability of a particular HPP treatment to achieve an inactivation target did differ between strains. We have previously reported the high efficacy (>5-log reduction) of HPP (500 MPa, 5–9 min) to inactivate *L. monocytogenes* ATCC 35152 in human milk (Liang et al., 2023). Other studies have described the high efficacy (> 5-log reduction) of HPP against other *L. monocytogenes* strains in human milk (Viazis et al., 2008; Jarzynka et al., 2021). HPP inactivation in these studies was likely enhanced at lower pressure (400–450 MPa) due to the ambient temperature at the initiation of processing (21 °C) which resulted in an elevated temperature at pressure due to adiabatic heating (31 °C).

None of the tested HPP treatments in this study were able to achieve the inactivation target (>5-log reduction) of *L. monocytogenes* in bovine milk. Previous studies have also demonstrated limited efficacy of HPP against *L. monocytogenes* in bovine milk at similar pressure/time/temperature combinations (Bull et al., 2005; García-Graells et al., 2000). This finding aligns with our previous work which demonstrated that more extreme HPP treatments (550 MPa, 12 min and 600 MPa, 8 and 12 min (with 30 °C processing temperature) were required to achieve > 5-log reductions of *L. monocytogenes* ATCC 35152 in bovine milk (Sykora et al., 2025). Many other studies have evaluated HPP treatment of bovine milk to inactivate *L. monocytogenes* and have confirmed the requirement of similar pressure treatments to achieve > 5-log reduction (Le Hurou-Luron et al., 2010; Shearer et al., 2010; Stratakosa et al., 2019).

UV-C treatment was also more effective in inactivating *L. monocytogenes* in human milk than in bovine milk. The 5-log reduction inactivation target for *L. monocytogenes* was achieved in human milk for both strains at UV-C doses of ≥ 6,000 J/L. In contrast, the highest dose used in this study (7,300 J/L) produced < 3-log reduction *of L. monocytogenes* in bovine milk. These results align with those of Liang et al. (2024), who reported that a UV-C dose of 4,000 J/L resulted in > 5-log reduction of *L. monocytogenes* ATCC 35152 in human milk as well as Barman et al. (2026), who reported that a UV-C dose of 12,000J/L was required to achieve a > 5-log reduction of the same strain in bovine milk.

UV-C antimicrobial efficacy is directly dependent on the ability of the light to penetrate the cell and cause DNA cross-linking. Access to the cell is highly depending on the optical properties of the food matrix. Differences in the opacity of human and bovine milk may impact the efficacy of the UV-C light to reduce *L. monocytogenes.* Optical properties of human and bovine milk were not measured in the current study; however, a companion study in our lab included an assessment of the optical properties of human and bovine milk samples (Supplementary Table 1). The refractive index of both milks was similar; however, the absorption coefficient, scattering coefficient were substantially lower for human milk than bovine milk and the UV transmittance values were higher in human milk than in bovine milk. Differences in the amount and structure of casein micelles and milk fat globules in the two milk types likely influence the optical properties of the two milk systems. These differences in optical properties support our observations that identical UV-C treatments would yield higher microbial reductions in human milk compared to bovine milk.

The current study identified differences in UV-C sensitivity between *L. monocytogenes* strains: ATCC 35152 was more sensitive than OSY-328. Significant differences in the UV-C sensitivity of *L. monocytogenes* strains treated in a buffer system were previously reported by (Gayán et al., 2015). Gayán et al. (2015) also reported comparable UV-C sensitivity for *L. monocytogenes* EGD-e and its isogenic Δ*sigB* mutant, suggesting that improved survival to UV-C treatments was not mediated by RNA polymerase sigma-B factor (σ^B^). These results further support the importance of strain selection and the use of comparable food matrixes for processing validation studies to support process preventive control or critical control point determinations for commercial processing (NACMCF, National Advisory Committee on Microbiological Criteria for Foods, 2010; Ceylan et al., 2021). Future investigations into mechanisms that promote increased UV-C resistance are warranted, particularly as UV-C treatments are adopted for an increasing number of food and beverage processing applications.

In addition to processing effects, this study also demonstrated extreme differences in *L. monocytogenes* behavior in bovine and human milk during extended refrigerated storage. *L. monocytogenes* strains were capable of relatively rapid and efficient growth in bovine milk, whereas *L. monocytogenes* populations declined throughout the storage period in human milk. Previous studies have demonstrated similar findings of the growth of *L. monocytogenes* in bovine milk. For example, Pitt et al. (1999) reported a 2-log increase in *L. monocytogenes* in raw bovine milk stored at 4 °C for 12 days. Pricope-Ciolacu et al. (2013) reported an increase of 3 log CFU/mL of *L. monocytogenes* in pasteurized bovine milk stored at 4 °C for 3 weeks. Castro et al. (2017) reported a similar increase of *L. monocytogenes* in raw bovine milk during storage at 6 °C for 14 days. Studies of the growth of *L. monocytogenes* in human milk are limited; however, Chen and Allen (2001) previously compared the behavior of *L. innocua* in raw and processed human and bovine milk with storage at 4 °C for up to 40 d. They observed a 2–3-log reduction of *L. innocua* in raw and pasteurized human milk stored at 4 °C for 40 d and a 2–3-log increase of the same inoculum in raw bovine milk within 20 d of refrigerated storage.

Milk processing interventions, particularly of human milk, had a significant impact on the behavior of *L. monocytogenes* during refrigerated storage. The anti-listerial activity of human milk was the strongest for milk processed by HoP and the weakest for milk processed by UV-C. As HoP can thermally denature and inactivate some antimicrobial compounds in milk, if the greater sensitivity of human milk observed is due to antimicrobial compounds, it would be due to those that retain structure and function following HoP treatment. HoP treatment reduces the concentration of structurally intact lactoferrin (by 98%), IgA (by 80%), IgG (by 56%), IgM (by 83%) in human milk, whereas HPP processing and UV-C treatments retain significantly higher concentrations of these proteins. In contrast, lysozyme is not significantly affected by any of the processing treatments (Liang et al., 2023; Liang et al., 2024; Tran et al., 2024). Therefore, it is unlikely that these bioactive proteins are predominantly responsible for the pattern of anti-listerial activity observed between the treated human milks. Recent research has also shown a significant decrease in monoglycerides in human milk after HoP (Escuder-Vieco et al., 2021); however, there is no published information on the impact of thermal processing on the GML concentration of human milk (which is much higher than bovine milk) – this has been previously identified as a gap in the literature (Vennard et al., 2024). Monoglyceride content of bovine milk has been shown to be stable to HPP treatments up to 900 MPa (Rodríguez-Alcalá et al., 2015); however, there seems to be no comparable information for monoglycerides or GML in HPP treated human milk (Pitino et al., 2023). The influence of UV-C treatment on GML is lacking, but substantial changes in saturated monoglyceride concentrations are not expected. Additional research is necessary to identify components or combinations of components responsible for the differential anti-listerial activity of processed human milk.

## Conclusion

The efficacy of HPP and UV-C treatments against *L. monocytogenes* significantly differed between human and bovine milk with both processing treatments being significantly more effective in human milk. Significant differences in the sensitivity of *L. monocytogenes* strains were identified in response to UV-C, but not HPP, processing treatments in both milk types with *L. monocytogenes* OSY-328 being more resistant than *L. monocytogenes* ATCC 35152. This result further substantiates the careful selection of strains and/or use multiple strains or cocktails for process validation studies to ensure food safety. Process schedules for milk products based on the inactivation of pathogens in bovine milk would be conservative for food safety purposes; however, these processes would be excessive for meeting safety goals for human milk which could lead to a loss of critical bioactive components that could have a significant impact on neonatal health. Human milk has a significant anti-listerial effect during extended storage which was retained after thermal pasteurization (HoP), but this effect is slightly muted after HPP processing and significantly impacted by UV-C processing. Future work is necessary to establish the influence of these processes on the antimicrobial components of human milk.

## Data Availability

The raw data supporting the conclusions of this article will be made available by the authors, without undue reservation.
