# Supplementary material for: Comparative inactivation of Listeria monocytogenes in human and bovine milk treated with high-pressure processing and UV-C treatment and potential growth during refrigerated shelf-life
Source: Front Microbiol. 2026 Apr 8;17:1774372. doi: 10.3389/fmicb.2026.1774372 (PMC13099874; doi:10.3389/fmicb.2026.1774372)
Supplement: Supplementary file 1 [file Table_1.DOCX]

Supplemental Table 1. Optical properties of raw human and bovine whole milk. Milk samples analyzed for optical properties were collected for a companion research project in our laboratory. Optical property data was provided by Dr. Ankit Patras, Tennessee State University. Methods used to collect optical property data are reported in Vahisht et al., 2022.

| **Optical properties** | **Whole human milk** | **Whole bovine milk** |
| --- | --- | --- |
| Absorption coefficient (cm^-1^) | 21.1 | 30.7 |
| Scattering coefficient (cm^-1^) | 1.92 | 53.57 |
| Refractive Index (at 254 nm) | 1.39 | 1.40 |
| UV transmittance (%/cm) | 1.48 E-19 | 1.97 E-29 |

**Reference**

Vahisht, P., B. Pendyala, A. Patras, V.V.S. Gopisetty, and R. Ravi. 2022. Design and efficiency evaluation of a mid-size serpentine Dean flow UV-C system for the processing of whole milk using computational fluid dynamics and biodosimetry. Journal of Food Engineering. 335: 111168. https://doi.org/10.1016/j.jfoodeng.2022.111168
